# FASTA/Q data compressors for MapReduce-Hadoop genomics: space and time savings made easy

**DOI:** 10.1186/s12859-021-04063-1

**Published:** 2021-03-22

**Authors:** Umberto Ferraro Petrillo, Francesco Palini, Giuseppe Cattaneo, Raffaele Giancarlo

**Affiliations:** 1 Dipartimento di Scienze Statistiche, Università di Roma - La Sapienza, Rome, Italy; 2 Dipartimento di Informatica, Università di Salerno, Fisciano, Italy; 3 Dipartimento di Matematica ed Informatica, Università di Palermo, Palermo, Italy

**Keywords:** MapReduce, Hadoop, Sequence analysis, Data compression

## Abstract

**Background:**

Storage of genomic data is a major cost for the Life Sciences, effectively addressed via specialized data compression methods. For the same reasons of abundance in data production, the use of Big Data technologies is seen as the future for genomic data storage and processing, with MapReduce-Hadoop as leaders. Somewhat surprisingly, none of the specialized FASTA/Q compressors is available within Hadoop. Indeed, their deployment there is not exactly immediate. Such a State of the Art is problematic.

**Results:**

We provide major advances in two different directions. Methodologically, we propose two general methods, with the corresponding software, that make very easy to deploy a specialized FASTA/Q compressor within MapReduce-Hadoop for processing files stored on the distributed Hadoop File System, with very little knowledge of Hadoop. Practically, we provide evidence that the deployment of those specialized compressors within Hadoop, not available so far, results in better space savings, and even in better execution times over compressed data, with respect to the use of generic compressors available in Hadoop, in particular for FASTQ files. Finally, we observe that these results hold also for the Apache Spark framework, when used to process FASTA/Q files stored on the Hadoop File System.

**Conclusions:**

Our Methods and the corresponding software substantially contribute to achieve space and time savings for the storage and processing of FASTA/Q files in Hadoop and Spark. Being our approach general, it is very likely that it can be applied also to FASTA/Q compression methods that will appear in the future.

**Availability:**

The software and the datasets are available at https://github.com/fpalini/fastdoopc

**Supplementary Information:**

The online version contains supplementary material available at 10.1186/s12859-021-04063-1.

## Background

Data Compression, as well as the associated techniques coming from Information Theory, has a long and very influential history for the storage and mining of biological data [1]. In recent years, it has received increasing attention via the proposal of novel specialized compressors, due to the facts that (a) storage costs have become quite significant given the massive amounts of data produced by HTS technologies (see [2] for an enlightening analysis, which is still valid [3, 4]); (b) generic compressors, even widely accepted leading methods, e.g. LZ4 [5], BZIP2 [6], are inadequate for the task of biological data compression. Good analytic reviews of the State of the Art are provided in [4, 7, 8], although no clear winner compressor has emerged. Here we concentrate on FASTA/Q files, since those formats, including unassembled genomic reads, have pervasive use in Bioinformatics.

### The relation between Big Data Technologies and FASTA/Q data compression in bioinformatics

Due to the same reasons of massive data production, the development and use of Big Data Technologies for Genomics and the Life Sciences, have been indicated as directions to be actively pursued [9], with MapReduce [10], Hadoop [11] and Spark [12] being the preferred ones [13]. This is not just a following of a “Big Data trend” that has proved successful in other fields of Science, since Bioinformatics solutions based on those techniques can be more effective than classic HPC ones, thanks to their scalability with available hardware and to their easiness of use. For later reference, it is worth pointing out that those technologies have “compression capabilities” via built-in generic data compressors, e.g., BZIP2 [6]. The corresponding software is referred to with the technical term Codec, where compression is coding and decompression is decoding. Moreover, although with quite some knowledge of those technologies, it is possible to add other compressors to Hadoop, i.e., additional Codecs. It is to be added that not all data compressors are amenable to a profitable incorporation due to the requirement of *splittable* compression: a file is divided into (un)compressed data blocks that can be compressed and decompressed separately granting in any case the integrity of the entire file. Indeed, processing files compressed using a non-splittable format is still possible under Hadoop, but at a cost of very long decompression times (data not shown but available upon request). Further discussion on those topics is in section “Preliminary”. We refer to the former category of data compressors as *splittable* Codecs.

Using the term *standard*, here and in what follows, to refer to a compressor that executes on a sequential machine, i.e., a PC, we observe that in order to make a compressor splittable, when its standard version is not, requires major code reorganization and rewriting.

Given the above discussion about Data Compression, it is rather surprising that the deployment of specialized compressors for biological data in Big Data technologies is episodic, in particular for FASTA/Q file formats, e.g., [14], that host a substantial part of genomic data.

### Methodological contributions

We provide two contributions for the deployment of standard specialised compressors for FASTA/Q files within MapReduce-Hadoop, together with the corresponding software.

- **Splittable Compressor Meta-Codec** When a standard compressor is splittable, we provide a method that facilitates its incorporation in Hadoop. Use of the software library associated to the method offers a substantial savings of programming time for a rather complicated task. Intuitively, the **Splittable Compressor Meta-Codec** performs a transformation of a standard splittable compressor in an Hadoop splittable Codec for that compressor.
- **Universal Compressor Meta-Codec** Independently of being splittable or not, as long as some mild assumptions in regard to input/output handling are satisfied, we provide a method to incorporate a data compressor in Hadoop, making it splittable. It is worth pointing out that the vast majority of standard specialized FASTA/Q compressors are not splittable. Again, intuitively, the **Universal Compressor Meta-Codec** performs a transformation of a standard compressor in an Hadoop splittable Codec for that compressor.

A few comments are in order. The **Splittable Compressor Meta-Codec** provides a template useful for accelerating and simplifying the development of specialized Hadoop Codecs. The **Universal Compressor Meta-Codec** allows to support in Hadoop any standard compressor with no programming at all, provided that it is usable as a command-line application. The first option has to be preferred when interested in achieving the best performance possible, at a cost of analyzing the internal format employed by files processed with that compressor and writing the required integration code. The second option allows to almost instantaneously support any command-line compressor, but at a cost of possibly reduced performance that we have measured to be negligible with respect to the direct use of the **Splittable Compressor Meta-Codec**. Both methods work also for Spark, when it uses the Hadoop File System.

It is also worth pointing out that the methods and software supporting both of the proposed Meta-Codecs leave unchanged the compression abilities of a standard compressor. Indeed, as explicitly discussed in the technical presentation given in Sections “General guidelines for the design of an Hadoop splittable Codec”, the transformations are limited to port the standard compressor to Hadoop, with the standard compression and decompression routines being treated as black boxes. This is particularly important for FASTQ files, where different fields are compressed with different methods even within the same standard compressor, e.g., [15]. Indeed, leading research in this area has studied thoroughly the various contributions to compression of FASTQ files given by its fields separately. The interested reader will find a good exposition of those studies in the Supplementary Material of [8, 15]. Therefore, it is important that the transformations proposed here do not alter the intrinsic abilities of any standard compressor to perform well (see Section 5 of the Additional file 1).

Finally, given the pace at which new standard specialized compressors are implemented, our methods can readily support the deployment of those future implementations in Hadoop.

For later use, we refer to the version of a standard compressor with the prefix HS when the incorporation in Hadoop has been made by using the **Splittable Compressor Meta-Codec** or an Hadoop splittable Codec is already available, e.g., LZ4 becomes HS_LZ4. Analogously, we use the prefix HU, when the **Universal Compressor Meta-Codec** has been used.

### Practical contributions

We provide experimental evidence that our methods are a major advance in dealing with massive data production in genomics within one of the Big Data technologies of choice. Indeed, for the **Universal Compressor Meta-Codec**, we show the following via an experimental comparative analysis involving a selection of specialized FASTA/Q compressors vs the generic compression Codecs already available in Hadoop.

- **Disk space savings** The size of the FASTA/Q files is significantly reduced with the use of specialized HU Codecs vs the generic HS ones available in Hadoop. Consequently, the cost of the hardware required to store them in the Hadoop File System is reduced.
- **Reading time savings** When using a specialized HU, the additional time required to decompress a FASTA/Q file in memory is counterbalanced by the much smaller amount of time required to load that file from the Hadoop File System. This results in a significant reduction of the overall reading time.
- **Network communication time overhead savings** The number of concurrent tasks required to process, in a distributed way, a FASTA/Q file compressed via an HU is greatly reduced, thus allowing for a significant reduction of the network communication time overhead required for the recombination of their outputs.

As for the **Splittable Compressor Meta-Codec**, we reach the same conclusions as above, but the experimentation is somewhat limited: the only standard specialized compressor for FASTA/Q files featuring a splittable format is DSRC [16]. Finally, disk space and reading time savings apply also to the Apache Spark framework, when used to process FASTA/Q files stored on the Hadoop File System.

## Methods

This section is organized as follows. Section “Preliminary” is dedicated to introduce some basic notions about Hadoop, useful for the presentation of our methods. Section “General guidelines for the design of an Hadoop splittable Codec” outlines some technical problems regarding the design of a splittable Codec for Hadoop, proposing our solutions. The last two section are dedicated to the description of our two Meta-Codecs.

### Preliminary

MapReduce is a programming paradigm for the development of algorithms able to process Big Data on a distributed system in an efficient and scalable way. It is based on the definition of a sequence of *map* and *reduce* functions that are executed, as *tasks*, on the nodes of a distributed system. Data communications between consecutive tasks is automatically handled by the underlying distributed computing framework, including the *shuffle* operation, required to move data from one node to another one of the distributed system.

In Section 1 of the Additional file 1 we provide more information about this topic, including Hadoop, one of the most popular MapReduce implementations. Here we limit ourselves to describe how files are stored in the Hadoop File System, i.e., HDFS.

When uploading a large file to HDFS (by default, larger than 128MB), it is automatically partitioned into several parts of equal size, where each part is referred to as *HDFS data block*. Each block is physically assigned to a Datanode, which are the nodes of the distributed system that execute map and reduce tasks.

For fault-tolerance reasons, HDFS data blocks can be replicated on several Datanodes according to a user-defined *replication factor*. This allows to process a HDFS data block even if the Datanode originally containing it becomes unavailable. By default, Hadoop assumes that each map task processes only the content of one particular HDFS data block. However, it may happen that, because of the aforementioned partitioning, a record to be analyzed by one map task is cut into two parts located in two different HDFS data blocks. We refer to these cases as *disalignments*.

This circumstance is managed by HDFS through the introduction of the *input split* concept or *split*, for short. It can be used, at the application level, to logically redefine the range of data to be processed by each map task, thus allowing a map task to process data found on HDFS data blocks different than the one it is processing.

#### Hadoop support for the input of compressed files

Currently, Hadoop supports two types of Codecs:

- *Stream-oriented.* Codecs in this class require that the whole file be available to each map task prior to decompressing it. For this reason, when a map task starts its execution, a request is issued to the other nodes of the cluster. As a result, all the parts of the file to be processed are collected from these nodes and merged into a single local file. This type of Codec can be developed by creating a new Java class implementing the standard Hadoop CompressionCodec interface.
- *Block-oriented.* Codecs in this class allow each map task to decompress only a portion of the input file, without requiring the remaining parts of it. They assume the compressed file to be logically split into data blocks, here referred to as *compressed data blocks*, where each of them can be decompressed independently of the others. Assuming the possibility of knowing the boundaries of each compressed data block, a map task can autonomously extract and decompress all the compressed data blocks existing in its HDFS data blocks. This type of Coded can be developed by creating a new Java class implementing the standard Hadoop SplittableCompressionCodec interface.

It is worth noting that the stream-oriented approach implies a significant computational overhead, as the same file is decompressed as many times as the number of map tasks processing it. It implies also a significant communication overhead, because the same file has to be replicated on each computational node running at least a map task. Finally, it may prevent a job from running at all because map tasks may not have enough memory to handle the decompression of the input file (e.g., when handling large files). For this reason, in this research, we focus on block-oriented Codecs, which are *de facto* splittable Codecs.

### General guidelines for the design of an Hadoop splittable Codec

Here we consider some problems that a programmer must face in order to obtain an Hadoop splittable compression Codec, offering solutions. We concentrate on genomic files, although those guidelines apply to any textual compressor. The solution we are seeking must not require any modification to the internal compression/decompression routines of the Codec to be supported. That is, the compression properties of a given compression algorithm must be preserved, so that previous benchmarking studies assessing how well a compressor does with respect to classes of datasets are not invalidated and users can make informed choices. For instance, for FASTQ files, compressors have been extensively benchmarked, also in regard to the contributions given to compression by the different fields of a FASTQ file, e.g., [8, 15]. Such a knowledge should be preserved.

There are two problems to face when extracting genomic sequences from a splittable compressed file. The first is about inferring the logical internal organization of the compressed file in regard to determine the relative position of the compressed data blocks. The second is in regard to the management of the possible disalignments existing between the physical partitioning of the file, as determined by HDFS, and the internal logical organization of the compressed file in compressed data blocks. In Section “Determining the internal structure of a compressed file” and in Section “Managing disalignments between compressed data blocks and HDFS data blocks”, respectively, these problems are described in detail and the solution we propose is presented, highlighting that the compression routines of a given compressor are not involved in our solution.

#### Determining the internal structure of a compressed file

A map task can extract and decompress the compressed data blocks existing in the HDFS data block it is analyzing only if it knows their size and relative positions. However, this information could be stored elsewhere (e.g., in the footer of the compressed file) or it could be encoded implicitly.

In the following, we provide a solution for efficiently dealing with the most frequent scenario, i.e., the one where the list of compressed data blocks is made explicitly available. We refer the interested reader to [6] for an example of a solution for encoding this list implicitly.

Explicit Representation. An explicit list of all the compressed data blocks existing in a compressed file is maintained in an auxiliary *index* data structure. This latter may either be located at the beginning or at the end of the file (e.g., DSRC [16]), or it can be saved in multiple copies along a file. In some other cases, this data structure can be saved in an external file complementing the compressed file.

In this case, the solution proposed here is to have one process to retrieve the index before processing the compressed file and send a copy to all nodes of the distributed system using the standard Hadoop Configuration class. Then, each computing node makes available this information to the map tasks that it runs, thus allowing them to determine the list and the relative position of the compressed data blocks in their HDFS data blocks.

#### Managing disalignments between compressed data blocks and HDFS data blocks

**Fig. 1:**
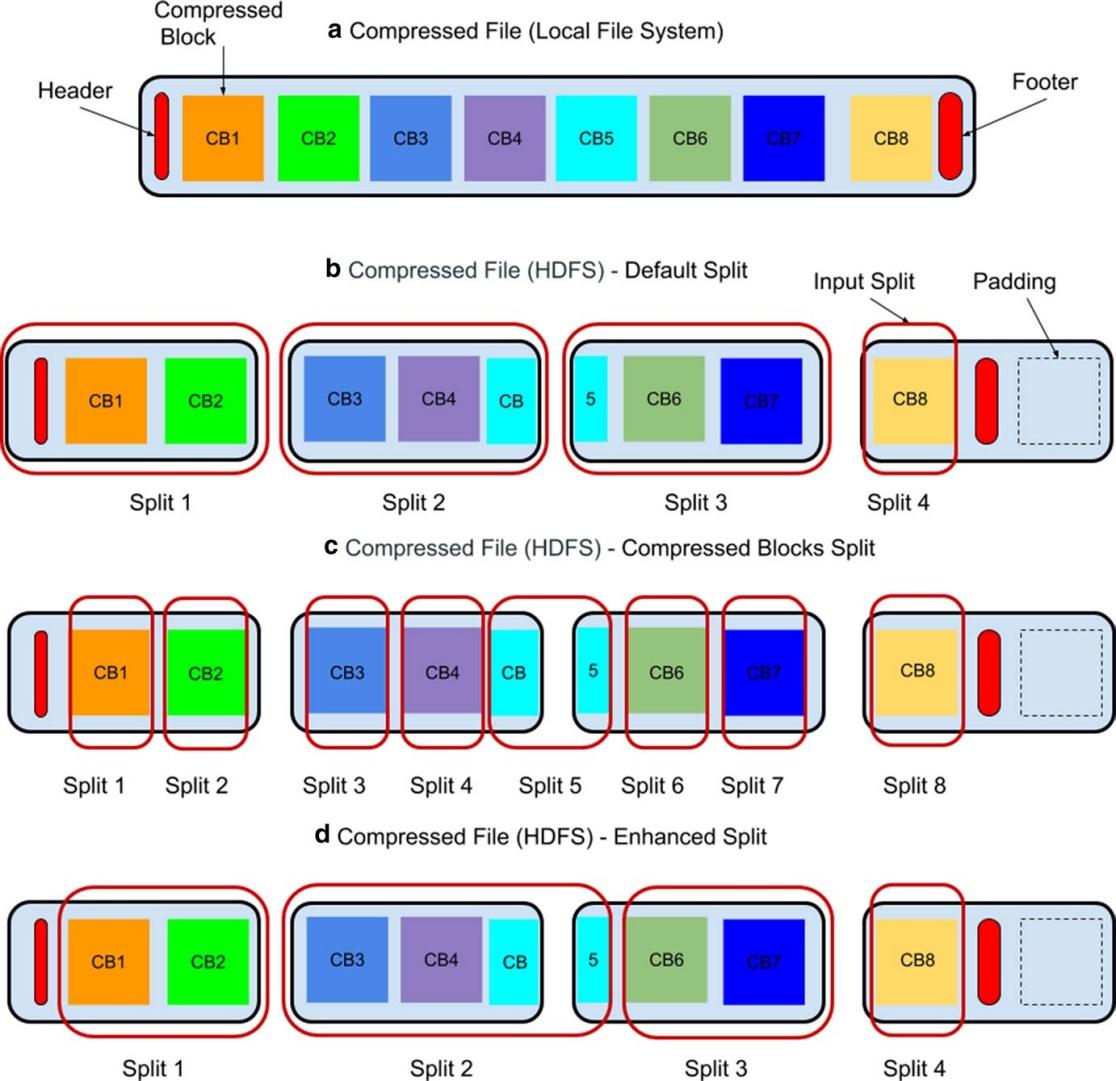
The layout of a block-oriented compressed data file when uploaded to HDFS. In the figure, **a** the original file includes an header, a footer and 8 compressed data blocks. **b** When uploaded to HDFS, it is partitioned into 4 HDFS data blocks. **c** As a result of the partitioning, the compressed data block labeled as *CB*5 is divided into two parts and assigned to two different HDFS data blocks. Using the *Compressed Block Split* strategy, each compressed data block is modeled as a distinct split. **d** Using the *Enhanced Split* strategy, several compressed data blocks are grouped into fewer input splits

When uploading a large compressed splittable file on HDFS, it is likely that several of its compressed data blocks would be broken into parts located on different HDFS data blocks, because of the partitioning strategy used by the distributed file system. An example of such a case is discussed in Fig. 1,with highlights given next. The file is initially stored as a whole on a local file system (Fig. 1a). If uploaded without specifying any splitting strategy, it would be partitioned into separate parts independently of the compressed data blocks, as pictured in Fig. 1b. This would imply a severe performance overhead when reading the content of compressed data blocks spawn across different parts because, in such a case, each node would be able to process its data blocks only after acquiring and decompressing together all the other parts of that file.

A first possible solution addressing such an overhead, and denoted as *Compressed Block Split* strategy, is to model as input splits all the compressed data blocks existing in a compressed file (see Fig. 1c). However, this strategy may imply also a performance overhead because the typical size of compressed data blocks is usually orders of magnitude smaller than those of the HDFS data blocks. Thus, the number of input splits would be much larger than the number of HDFS data blocks.

A more efficient solution, here denoted as *Enhanced Split* strategy, is to fit several compressed data blocks into the same Hadoop input split and, then, have each map task query a local index listing the offset of all the single compressed data blocks existing in a split (see Fig. 1d). At this point, when processing compressed data blocks in a split, two cases may occur:

- **standard case** the compressed data block is entirely contained in a single HDFS data block. In such a circumstance, it is retrieved using the information contained in the index and, then, decompressed using the considered Codec.
- **exceptional case** the compressed data block is physically divided by HDFS into two parts, $p_{1}$ and $p_{2}$. These parts are located on two HDFS data blocks but are assigned to the same input split. In such a case, a copy of $p_{2}$ is automatically pulled from the Datanode holding it. Then, $p_{1}$ and $p_{2}$ are properly concatenated to obtain *p*. The resulting compressed data block is decompressed using the Codec decompression function.

### The architecture of the splittable compressor Meta-Codec

This Meta-Codec consists of a library of abstract Java classes and interfaces implementing a standard Hadoop splittable Codec for the compression of FASTA/Q files, but with empty compression/decompression routines.

Its architecture is based on a specialization of the generic compressors and decompressors interface coming with Hadoop and targeting block-based Codecs. It offers the possibility to automatically assemble a compressed file as a set of compressed data blocks while maintaining their index using an explicit representation, as described in Section “Determining the internal structure of a compressed file”. In addition, the compressed data blocks are organized according to the Enhanced Split strategy (see Section “Managing disalignments between compressed data blocks and HDFS data blocks”). Compression/decompression occurs using the original routines available with the target Codec, without requiring any modification to their internal implementation. Also the creation of the compressed data blocks index is automatically managed by our Meta-Codec, which also provides the ability to share the content of the index with all nodes of an Hadoop distributed system so to allow for each node to know the exact boundaries of the compressed data blocks it has to process. Additional details regarding the architecture of this Meta-Codec are given in Fig. 1 of the Additional file 1. Here we limit ourselves to mention that it includes the following Java classes.

- CodecInputFormat. It fetches the list of compressed data blocks existing in a compressed file and sends it to all the nodes of an Hadoop cluster together with the instructions required for their decompression. Then, it defines the input splits as containers of compressed data blocks. These operations are compressor-dependent and require the implementation of several abstract methods like extractMetadata, to extract the metadata from the input file, and getDataPosition, to point to the starting address of the first compressed data block.
- NativeSplittableCodec. Assuming the compression/decompression routines for a particular Codec are available as a standard library installed on the underlying operating system, it simplifies its integration in the Codec under development.
- CodecInputStream. It reads the compressed data blocks existing in a HDFS data block, according to the input split strategy defined by the CodecInputFormat. The compressed data blocks are decompressed on-the-fly by invoking the decompression function of the considered compressor and returned to the main application. Some of these operations are compressor-dependent and require the implementation of the setParameters abstract method. This method is used to pass to the Codec the command-line parameters required by the compressor, e.g execution flags, in order to correctly decompress the compressed data blocks.
- CodecDecompressor. It decompresses the compressed data blocks given by the CodecInputStream. It requires the implementation of the decompress abstract method.
- NativeCodecDecompressor. It decompresses the compressed data blocks given by the CodecInputStream. It requires the implementation of the decompress method through the native interface.

### The architecture of the Universal Compressor Meta-Codec

This Meta-Codec is a software component able to automatically expose as a HU splittable Codec the compression/decompression routines offered by a given standard compressor. As opposed to the **Splittable Compressor Meta-Codec**, requiring some programming, it works as a ready-to-use black box, since the only information it needs is the set of command lines to be used for compressing and for decompressing an input file by means of a standard compressor. This implies that no modification is performed on the internal routines of the given compressor.

Assuming there is an input file to compress in a splittable way, this method works by splitting the file into uncompressed data blocks and, then, compressing each uncompressed data block using an external compression application according to the command line given at configuration time. As for the **Splittable Compressor Meta-Codec**, compressed data blocks are organized following the Enhanced Split strategy (see Section “Managing disalignments between compressed data blocks and HDFS data blocks”).

The resulting file uses an index for the explicit representation of the compressed data blocks existing therein (see Section “Determining the internal structure of a compressed file”) based on the following format.

- **compression_format**: A unique id number telling the Codec format used for this file.
- **compressed_data_blocks_number**: Number of compressed data blocks existing in the file.
- **blocks_sizes_list**: List of the size of all the compressed data blocks included in the file.
- **uncompressed_block_size**: The size of the data structure used for decompressing the compressed data blocks.

The decompression is achieved by exploiting the information contained in the aforementioned index.

The usage of this Meta-Codec assumes the possibility of parking as files on a local device the content of the (un)compressed data blocks to process. For efficiency reasons, these are saved on the local RAM disk, a virtual device usable as a disk but with the same performance of memory.

**Fig. 2:**
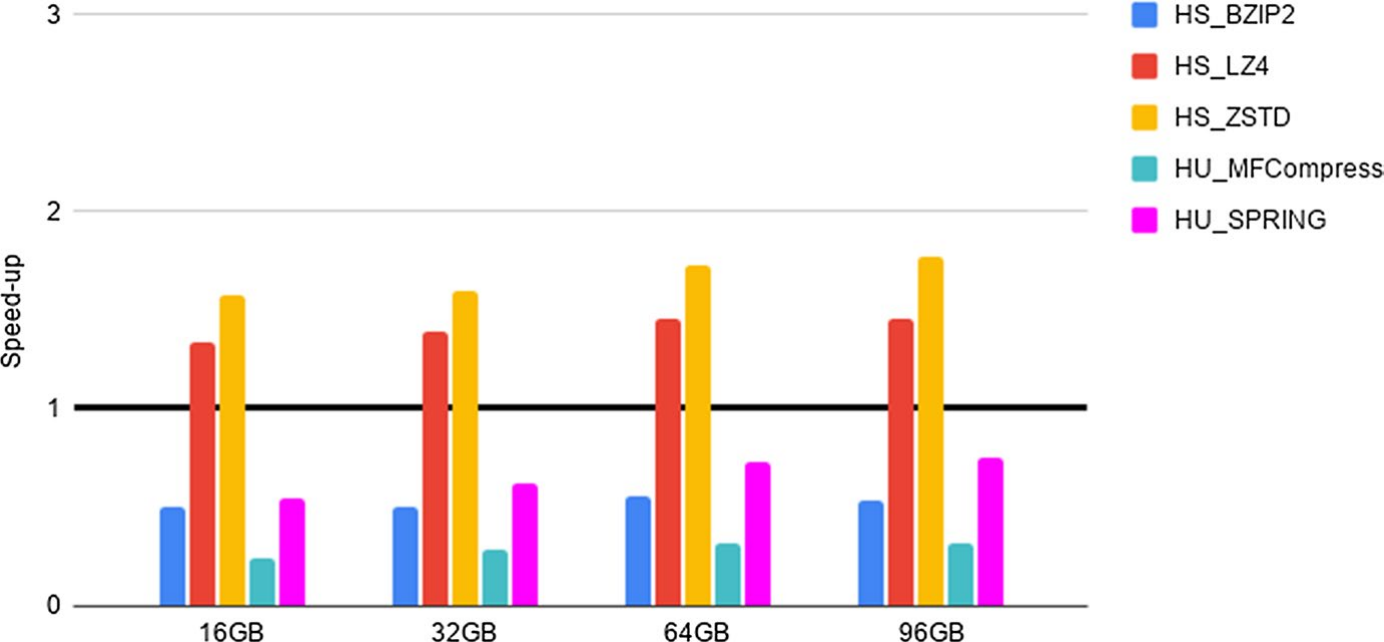
Experiment 2-Reading time savings. Type 1 datasets FASTA files. HDFS reading time speed-up when considering files of increasing size and different compressors. The speed-up has been evaluated as specified in the main text. The tick line denoted equal time performance of “compressed vs uncompressed ” execution. Values greater than 1 denote a speed-up, while values smaller than 1 denote a slow-down. The abscissa denotes file size and the speed-up of each compressor is denoted by a bar, with color as indicated in the top right of the figure

The Java classes for this Meta-Codec, shown in Fig. 2 of the Additional file 1, are the following.

- Algo. Contains the command-line instructions of a particular compressor, defined through the configuration file.
- UniversalCodec. Contains fields and methods for managing data compression and decompression.
- UniversalInputFormat. Extends the CodecInputFormat class, implementing the methods according to the compressed file structure.
- UniversalDecompressor. Extends the CodecDecompressor class, implementing the method decompress, according to the command-line commands of the Algo object.

### Experimental setting

#### Choice of compression Codecs: standard specialized or available in Hadoop

For our experiments, all the standard splittable general-purpose compression Codecs available with Hadoop have been considered: BZIP2 [6], LZ4 [5] and ZSTD [17].

As for the specialized FASTA/Q files compressors, we have developed a set of compression Codecs based on SPRING [15], DSRC [16], Fqzcomp [18], MFCompress [19]. These have been chosen, with independent experiments, as they cover the range of possibilities in terms of the trade-off compression and time. A list of all these Codecs is reported in Table 1, with their relevant features for this research.

**Table 1:** List of splittable Codecs considered in our experiments

Compressor	Input FormatType	Implementation
BZIP2 [6]	Any file	HS
LZ4 [5]	Any file	HS
ZSTD [17]	Any file	HS
DSRC [16]	FASTQ files	HS/HU
Fqzcomp [18]	FASTQ files	HU
MFCompress [19]	FASTA files	HU
SPRING [15]	FASTA/Q files	HU

For each splittable Codec, it is reported: (1) the originating compressor; (2) the input format it supports; (3) whether or not it has been developed using our **Splittable Compressor Meta-Codec** (HS) or our **Universal Compressor Meta-Codec** (HU) or directly supported (HS)

It is to be remarked that while the general purpose compressors have been designed to compress well and be fast in compression/decompression times, the specialized ones are not so uniform with respect to this design criteria. For instance, HU_SPRING compresses very well, but it is very slow in compression/decompression times, while HU_DSRC offers a good balance of those aspects. To place every compressor at a peer, we use their default settings.

#### Datasets

We have used for our experiments two types of datasets. Both of them contain files that are collections of reads and therefore are good representatives of files that are the end product of HTS technologies. Details regarding those datasets are in Section 4 of the Additional file 1.

The first type of dataset, here referred to as **type 1 datasets**, is a collection of FASTQ and FASTA files, of different sizes. The FASTQ files contain a set of reads extracted uniformly and at random from a collection of genomic sequences coming from the Pinus Taeda genome [20], while the FASTA files contain a set of reads extracted uniformly and at random from a collection of genomic sequences coming from the Human genome [21]. We have chosen these datasets because they are so large to represent a relevant benchmark for the type of experiment we were interested in, allowing us to assess changes in performance of the compressors according to a predetermined file size.

The second type of dataset, here referred to as **type 2 datasets**, is a collection of FASTQ files, providing different coverages of *H.sapiens*. They have been already used to benchmark SPRING and we have followed the same instructions available in the Supplementary Material of [15]. We have chosen these datasets because they allow us to measure the potential performance advantages coming from the processing of files with increasing degree of redundancy.

#### Hardware

The testing platform used for our experiments is a 9 nodes Linux-based Hadoop cluster, with one node acting as *resource manager* and the remaining nodes being used as workers. Each node of this cluster is equipped with two 8-core Intel Xeon E3-12@2.70 GHz processor and 32GB of RAM. Moreover, each node has a 200 GB virtual disk reserved to HDFS, for an overall capacity of about 1.6 TB. All the experiments have been performed using the Hadoop 3.1.1 software distribution.

## Results

**Fig. 3:**
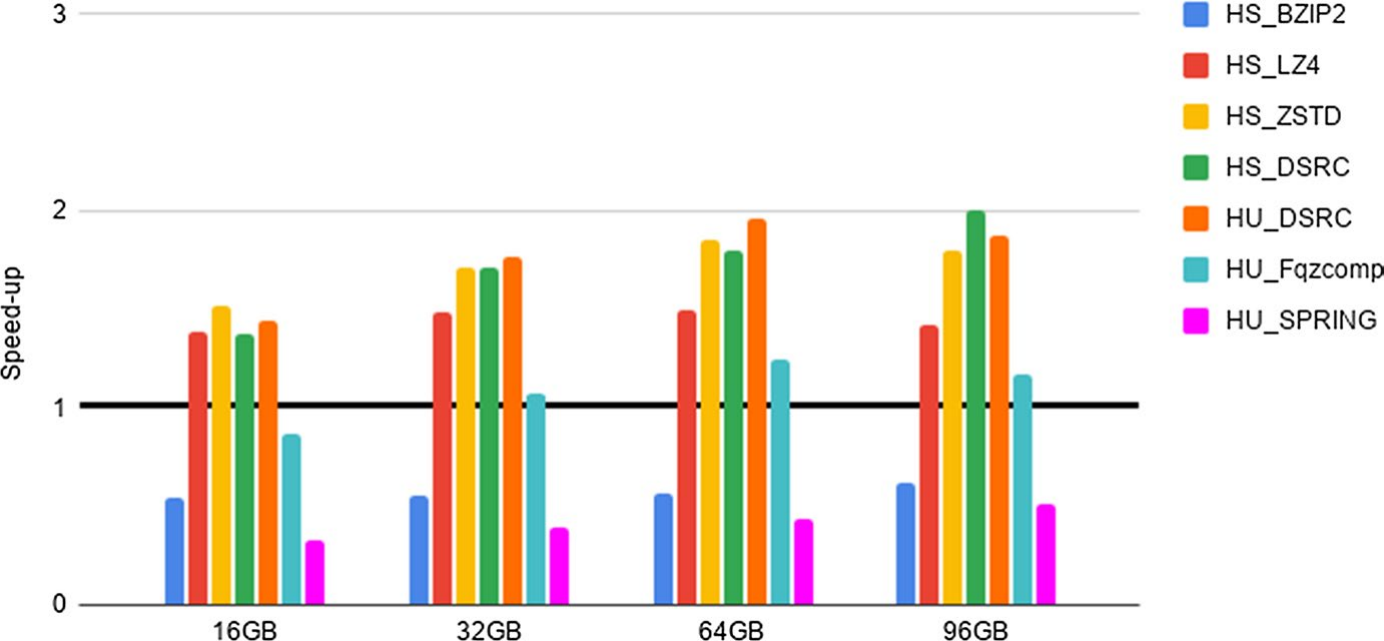
Experiment 2-Reading time savings. Type 1 datasets FASTQ files. The Figure Legend is as in Fig. 2

**Fig. 4:**
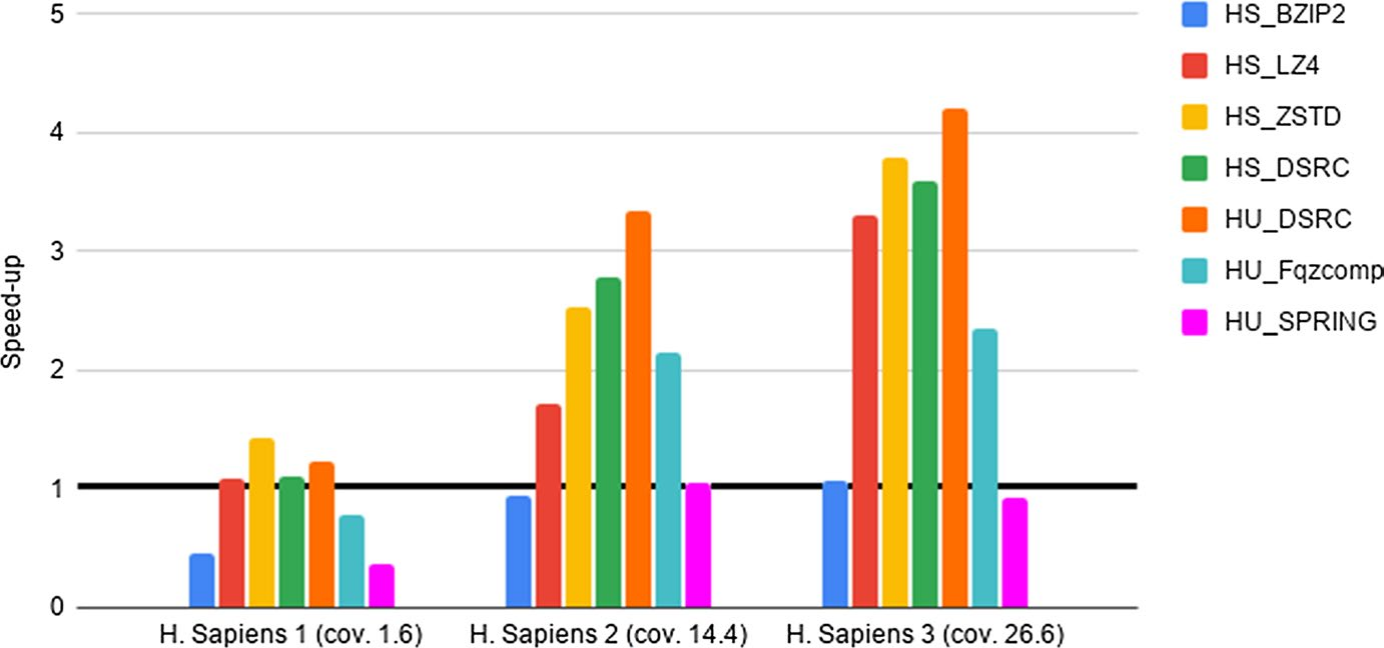
Experiment 2-Reading time savings. Type 2 datasets FASTQ files. The Figure Legend is analogous to the one in Fig. 2

**Fig. 5:**
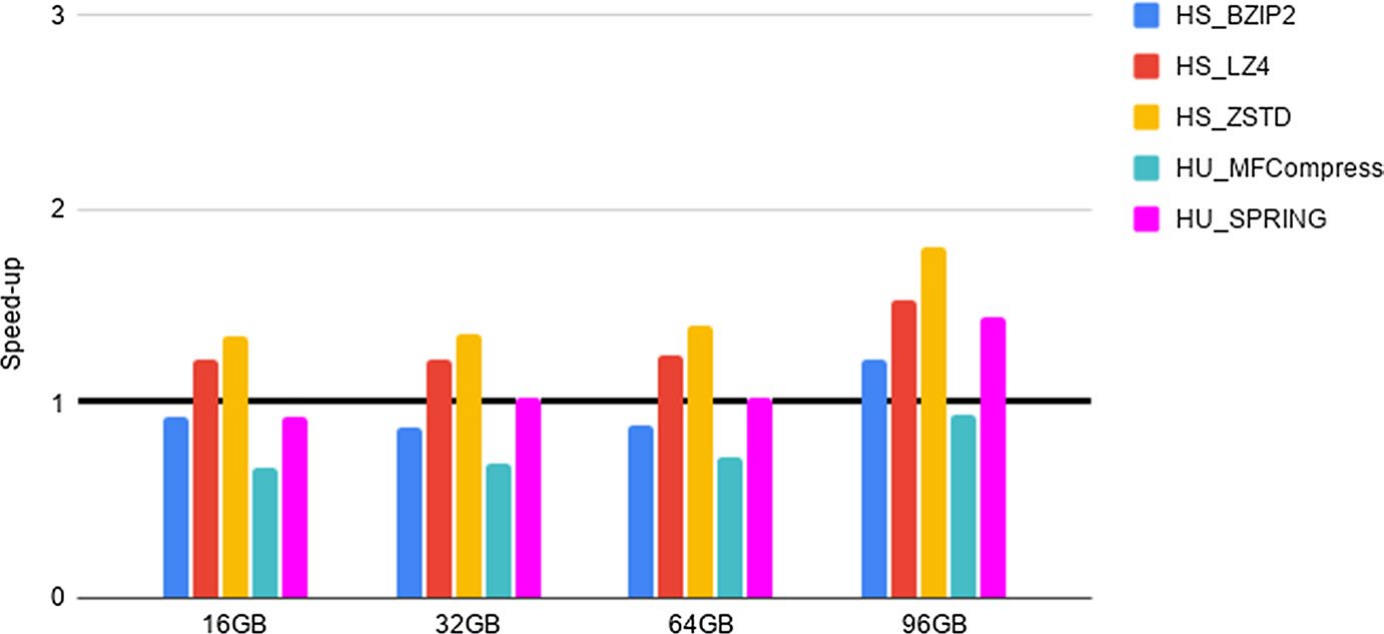
Experiment 3-Network overhead savings. Type 1 datasets FASTA files. Execution time speed-up of benchmarking task 2, measured when considering files of increasing size and different compressors. Speed-up has been evaluated as specified in the main text. The tick line denoted equal time performance of “compressed vs uncompressed” execution. Values greater than 1 denote a speed-up, while values smaller than 1 denote a slow-down

**Table 2:** Experiment 1-Space Savings. Type 1 dataset FASTA files

Dataset	HS_BZIP2 (%)	HS_LZ4 (%)	HS_ZSTD (%)	HU_MFCompress (%)	HU_SPRING (%)
16G	81	55	76	85	86
32G	82	55	76	85	86
64G	82	55	76	85	87
96G	82	55	76	85	87

Space savings (in percentage) and in terms of HDFS blocks, computed as specified in the main text. Files of increasing size are listed on the rows, while general-purpose and specialized compression Codecs are listed on the columns

**Table 3:** Experiment 1-Space Savings

Dataset	HS_BZIP2 (%)	HS_LZ4 (%)	HS_ZSTD (%)	HU_DSRC (%)	HS_DSRC (%)	HU_Fqzcomp (%)	HU_SPRING (%)
16G	81	53	74	83	84	84	84
32G	81	54	75	83	84	84	84
64G	81	53	75	82	84	84	84
96G	81	53	75	84	84	86	87

Type 1 dataset FASTQ files. The Table Legend is as in Table 2

As a preliminary step, we provide evidence that the HS and HU Codecs imported in Hadoop via our methods preserve the compression properties of the stand-alone compressors, as required in Section “General guidelines for the design of an Hadoop splittable Codec”. Such an experiment, together with the results, is presented and briefly discussed in Section 5 of the Additional file 1. Then, in order to quantify the advantages of deploying FASTA/Q Codecs in Hadoop via our methods, we perform the experiments detailed below, that we briefly justify next. The intent of Experiments 1-3 is to provide evidence of the space and time performance advantages deriving from the adoption of specialized FASTA/Q compressors within MapReduce-Hadoop. Finally, Experiment 4 is provided for completeness, since it assesses the possible space compression loss, referred in what follows as overhead, due to the usage of a HS and/or HU Codec against the usage of a same stand-alone compressor, i.e., when executed in a non distributed setting.

**Fig. 6:**
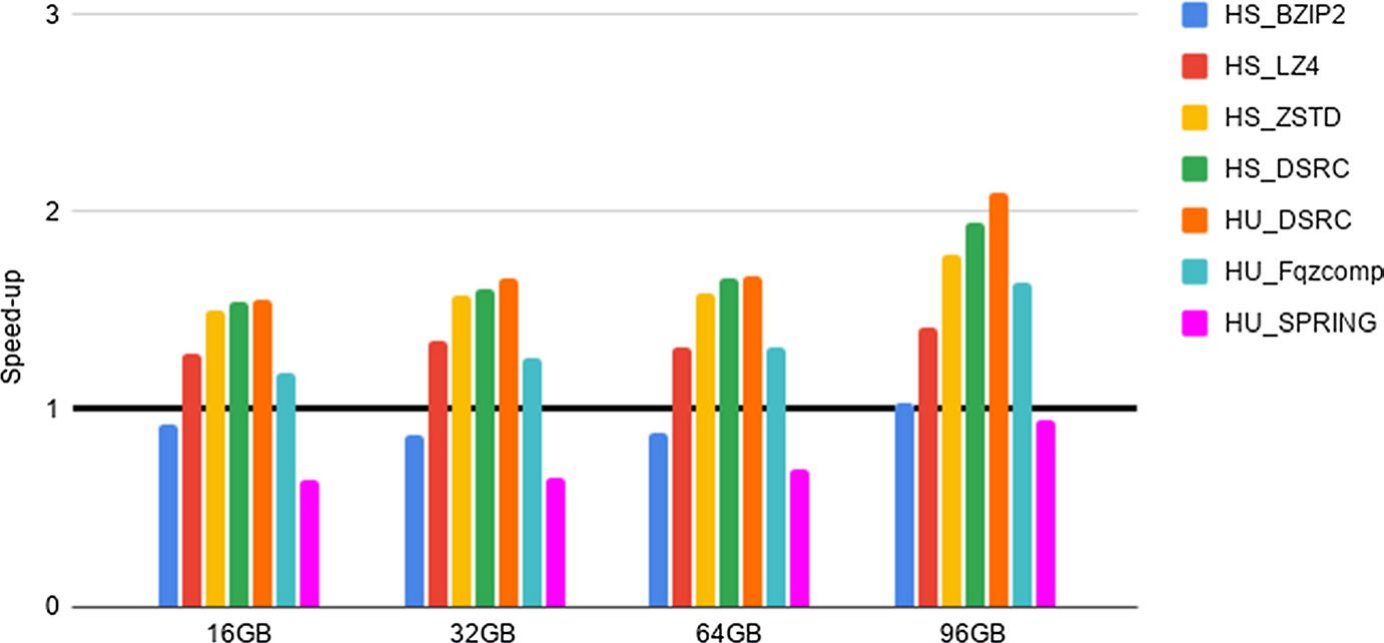
**Experiment 3-Network overhead savings. Type 2 datasets FASTQ files.** The Figure Legend is as in Fig. 5

**Fig. 7:**
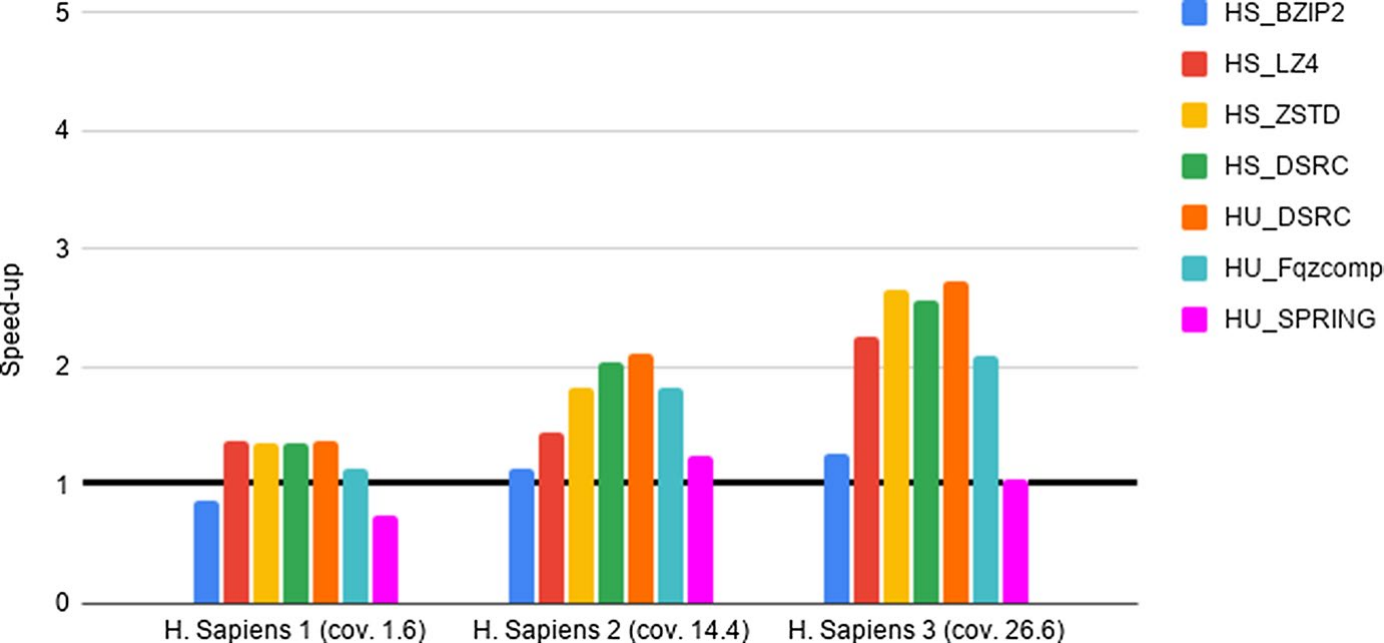
Experiment 3-Network overhead savings. Type 1 datasets FASTQ files. The Figure Legend is as in Fig. 5

- **Experiment 1: An assessment of disk space savings** The aim here is to determine the possible disk space savings achievable thanks to the adoption of a specialized HU or HS Codec, when storing FASTA/FASTQ files on the Hadoop HDFS distributed file system, with respect to the usage of general-purpose HS Codecs available in Hadoop. Space savings (in percentage), in terms of HDFS data blocks, has been computed according to the following formula. Let *F* be an input genomic file and $F'$ its compressed splittable representation, then space savings is $1 - \frac{(\text {size of F' in HDFS blocks )}}{ (\text {size of F in HDFS blocks})}$. The size of each HDFS data block is 128 MB. The results of this experiment are reported in Tables 2, 3, 4.
- **Experiment 2: An assessment of reading times savings** The aim here is to determine if there is a positive trade-off between the time saved thanks to the smaller amount of data to read from HDFS and the cost to be paid for reading and unpacking compressed FASTA/Q files, once compressed with an HS or an HU Codec. Following the methodology used in [22], this experiment is implemented by benchmarking a very simple Hadoop application. It runs only map tasks whose goal is to count the number of occurrences of the letters $\{A,C,G,T,N\}$ in the input sequences, without producing any output. That is, the application spends most of its time reading data from HDFS. In what follows, we refer to such a task as benchmarking task 1. Speed-up has been evaluated by dividing the overall execution time of experiments run on each uncompressed file with respect to the overall execution time of the same experiment, but run on the same compressed file. The results are reported in Figs. 2, 3, 4.
- **Experiment 3: An assessment of network communication time overhead savings** The aim here is to establish if the smaller amount of network traffic due to the reduced number of map tasks needed to process a FASTA/Q file compressed with an HS or HU Codec has a beneficial effect on the overall shuffle time of an application, compared to the case where the input file is uncompressed. This experiment is implemented by benchmarking an application where each map task counts the number of occurrences of the letters $\{A,C,G,T,N\}$, in each of the sequences read from an input file. Once finished, the map task emits, as output, the overall count for each of the considered sequences. The reduce tasks gather and aggregate the output of all map tasks, and print on output the overall number of occurrences of each distinct letter. That is, the execution of this experiment requires a communication activity between map and reduce tasks that is proportional to the number of map tasks being used. In what follows, we refer to such a task as benchmarking task 2. Speed-up has been evaluated by dividing the overall execution time of experiments run on each uncompressed file with respect to the overall execution time of the same experiment, but run on the same compressed file. The results are reported in Figs. 5–7.
- **Experiment 4: An assessment of the compression loss due to the usage of our HS and HU Codecs against the stand-alone methods** The aim here is to determine the possible disk space overhead introduced by the usage of a target Codec via our HS and HU Codecs, when storing FASTA/FASTQ files on the Hadoop HDFS distributed file system, with respect to the space usage needed to store the same files on a non-distributed setting using the original version of the same Codec. The overhead has been computed as follows. Let *CS* be a file compressed using a stand-alone compressor and *CH* be the same file compressed using its corresponding HS or HU Codecs, the file size overhead is $\frac{(\text {size of CH in bytes})}{(\text {size of CS in bytes})}-1$. The results of this experiment are reported in Tables 5, 6, 7.

**Table 4:** Experiment 1-Space Savings: Type 2 datasets FASTQ files

Dataset	HS_BZIP2 (%)	HS_LZ4 (%)	HS_ZSTD (%)	HS_DSRC (%)	HU_DSRC (%)	HU_Fqzcomp (%)	HU_SPRING (%)
H. Sapiens 1 (cov. 1.6x)	75	45	67	78	78	81	81
H. Sapiens 2 (cov. 14.4x)	74	41	65	77	77	80	80
H. Sapiens 3 (cov. 26.6x)	86	62	80	86	86	89	89

The Table Legend is analogous to the one in Table 2

**Table 5:** Experiment 4-File size overhead

Dataset	BZIP2	LZ4	ZSTD	MFCompress	SPRING
	SA vs HS	SA vs HS	SA vs HS	SA vs HU	SA vs HU
16GB	$\sim 0\%$	$\sim 0\%$	$\sim 0\%$	$\sim 4.35\%$	$\sim 46.67\%$
32GB	$\sim 0\%$	$\sim 0\%$	$\sim 0\%$	$\sim 9.09\%$	$\sim 59.26\%$
64GB	$\sim 0\%$	$\sim 0\%$	$\sim 0\%$	$\sim 7.95\%$	$\sim 95.45\%$
96GB	$\sim 0\%$	$\sim 0\%$	$\sim 0\%$	$\sim 7.63\%$	$\sim 134.55\%$

Type 1 datasets FASTA files: Space overhead (in percentage), computed as specified in the main text, introduced by compression Codecs encapsulated in the HU and in the HS Codecs vs their (SA) stand-alone versions (on the columns), when compressing input files of increasing size (on the rows)

## Discussion

### Experiment 1: Specialized compression yields significant disk space savings on Hadoop.

With reference to Tables 2, 3, 4, it is evident the ability of the specialized HU and HS Codecs, i.e. the ones that have been imported in Hadoop using our methods, to reach space savings significantly better than that of generic HS Codecs already available in Hadoop. This is witnessed by the much smaller number of HDFS data blocks needed to store a distributed compressed representation of each file, with respect to uncompressed files. In particular, all specialized codecs exhibit better performance than the non-specialized ones, with HU_Fqzcomp and HU_SPRING somewhat better than HS_DSRC and HU_DSRC.

**Table 6:** Experiment 4-File size overhead: Type 1 datasets FASTQ files: The Table Legend is as in Table 5

Dataset	BZIP2	LZ4	ZSTD	DSRC	DSRC	Fqzcomp	SPRING
	SA vs HS	SA vs HS	SA vs HS	SA vs HS	SA vs HU	SA vs HU	SA vs HU
16GB	$\sim 0\%$	$\sim 0\%$	$\sim 0\%$	$\sim 0\%$	$\sim 0\%$	$\sim 4.55\%$	$\sim 10.53\%$
32GB	$\sim 0\%$	$\sim 0\%$	$\sim 0\%$	$\sim 0\%$	$\sim 0\%$	$\sim 2.27\%$	$\sim 10.53\%$
64GB	$\sim 0\%$	$\sim 0\%$	$\sim 0\%$	$\sim 0\%$	$\sim 0\%$	$\sim 1.12\%$	$\sim 16.44\%$
96GB	$\sim 0\%$	$\sim 0\%$	$\sim 0\%$	$\sim 0\%$	$\sim 0\%$	$\sim 1.49\%$	$\sim 11.01\%$

It is to be noted that those latter methods yield nearly identical compression results. This is an important indication that our Universal Meta-Codec is effective and worthwhile. Indeed, the development of HS_DSRC took non trivial programming skills as well as several days of work, while the development of HU_DSRC took few minutes to be developed and no programming.

### Experiment 2: a careful use of specialized compression yields significant reading-times savings on Hadoop for FASTQ files.

Compression may significantly reduce the amount of time required to read data from an external device, as long as the decompression process is “fast”. Such a trade-off is well known for generic standard compressors. Here we study it in regard to HS and HU specialized Codecs. Our experiments indicate the following.

- **FASTA performance for type 1 datasets**. We notice here that the trade-off between compression space efficiency and decompression times matters. On a side, we observe from Table 2 that the usage of the specialized Codecs leads to a better compression with respect to non specialized Codecs. However, this advantage is canceled out by the much slower decompression routines of specialized Codecs, as witnessed by the results reported in Fig. 2. In order to support such a conclusion, it is worth recalling that the benchmarking task 1 consists of reading compressed files and then of decompressing them. Consequently, the usage of specialized codecs does not bring a positive speed-up in this setting. Our experiments, however, provide the following novel and useful guidelines. When fast reading time is critical, it is recommended to use HU_ZSTD as it allows for a significant time performance speed-up while guaranteeing consistent space savings. When space is important, while accounting for reading time also, HU_SPRING is better than HU_MFCompress.
- **FASTQ performance for type 1 datasets and type 2 datasets**. As well illustrated by the results in Figs. 3, 4, HS_DSRC and HU_DSRC are among the top performers. Indeed, their reading and decompression time performance is better or quite close to that of a highly engineered generic compressor such as ZSTD. This is mostly due to their significant compression ability and to their very fast decompression routines. Moreover, as the dataset size grows and/or its redundancy increases, the performance of those two methods gets better and better. To explain this, consider that when managing the 16G input file, HS_DSRC and HU_DSRC return a number of HDFS data blocks to process that is smaller than the number of available processing cores. So, not all the available processing capability of the cluster is exploited. When the size of the input increases to 32G, the number of HDFS data blocks gets larger and allows to use all the available processing cores, thus resulting in an improved overall efficiency. The novel indication that we get from our experiments is that HS_DSRC and HU_DSRC are definitely the methods of choice for FASTQ files, when space is at a premium and reading time from HDFS is important.

It is to be noted that also this experiment confirms the validity and convenience of our Universal Meta-Codec, since the reading time speed-up of HS_DSRC and HU_DSRC are very close on all of the experiments we have performed.

### Experiment 3: a careful use of specialized compression yields significant network communication cost on Hadoop for FASTA/Q files.

When considering more complex Hadoop applications, such as our second benchmarking task, we notice that the usage of specialized Codecs allows for a significant time performance speed-up (see Figs. 5, 6, 7). This is mostly due to the beneficial effect on the network traffic flowing from Hadoop map tasks to Hadoop reduce tasks thanks to the reduced amount of HDFS data blocks to process. In particular, our experiments suggest the following evaluation.

- **FASTA performance for type 1 datasets**. According to the experimental results depicted in Fig. 5, HU_SPRING, while preserving its superior compression performance with respect to the other compressors, can also provide some speed-up (or at least, no sensible performance degradation) in terms of network traffic savings. This latter, then, results in an overall time savings.
- **FASTQ performance for type 1 datasets and type 2 datasets**. According to the experimental results depicted in Figs. 6, 7, HS_DSRC and HU_DSRC are consistently among the top performers, for the same reasons outlined in the discussion of the previous experiment.

Finally, also this experiment supports the validity of our Universal Codec.

### Experiment 4: The compression loss of distributed HS and HU Codecs with respect to the stand-alone ones is minimal, provided those latter are amenable to splittability.

**Table 7:** Experiment 4-File size overhead: Type 2 datasets FASTQ files: The Table Legend is as in Table 5

Dataset	BZIP2	LZ4	ZSTD	DSRC	DSRC	Fqzcomp	SPRING
	SA vs HS	SA vs HS	SA vs HS	SA vs HS	SA vs HU	SA vs HU	SA vs HU
H. Sapiens 1 (cov. 1.6x)	$\sim 0\%$	$\sim 0\%$	$\sim 0\%$	$\sim 0\%$	$\sim 0\%$	$\sim 4.76\%$	$\sim 10.00\%$
H. Sapiens 2 (cov. 14.4x)	$\sim 0\%$	$\sim 0\%$	$\sim 0\%$	$\sim 0\%$	$\sim 0\%$	$\sim 1.41\%$	$\sim 43.71\%$
H. Sapiens 3 (cov. 26.6x)	$\sim 0\%$	$\sim 0\%$	$\sim 0\%$	$\sim 0\%$	$\sim 0\%$	$\sim 1.41\%$	$\sim 154.43\%$

With reference to Tables 5, 6, 7, it is clear that the space overhead introduced by our HS and HU Codecs to bring any standard compressor to Hadoop is, in most cases, negligible. This is particularly true for Codecs that are natively splittable or are, at some degree, amenable to splittability. However, “global compression strategies” are not amenable to splittability, as well illustrated by SPRING. Indeed, its compression algorithm first reorders reads so that they are approximately ordered to their position in the genome and, then, the ordered reads are used to assemble a reference genome that is finally compressed, while removing duplicate reads. Indeed, such an approach is expected to work very well when processing very large and redundant files. Conversely, its compression efficiency is not so effective, when working separately on each piece of a partitioned file, as HDFS requires. In particular, with the aid of

**Table 8:** Datasets Compressibility-SPRING: Type 1 datasets: Space savings (in percentage) of SPRING when processing files of increasing size (on rows)

Dataset	FASTA (%)	FASTQ (%)
16GB	90	86
32GB	91	86
64GB	93	86
96GB	94	86

Let *F* be an input genomic file and $F'$ be the same file compressed using SPRING as stand-alone application, the space saving is computed as $1 - \frac{\text {(size of F' in bytes)}}{\text {(size of F in bytes)}}$

**Table 9:** Datasets Compressibility-SPRING: Type 2 datasets: The Table Legend is as in Table 8

Dataset	FASTQ (%)
H. Sapiens 1 (cov. 1.6x)	83
H. Sapiens 2 (cov. 14.4x)	86
H. Sapiens 3 (cov. 26.6x)	96

Tables 8, 9, it is evident that the overhead introduced by HU_SPRING, with respect to its stand-alone version, grows with the compressibility of the files. Quite remarkably, despite this significant space overhead, HU_SPRING is still among the best for compression over Hadoop (see the discussion regarding **Experiment 1** again).

It is also of interest to point out that although BZIP is based on the Burrows and Wheeler transform [23], it applies such a transform to small chunks of a file (900 Kb each). Therefore, although in principle compression “a la Burrows and Wheeler” could be implemented as a global strategy, BZIP compression is “local” since it works on small pieces of a file separately. As a consequence, its distributed version has virtually no overhead with respect to the stand-alone one.

## Conclusions

We have provided two general methods that can be used to transform standard FASTA/Q data compression programs into Hadoop splittable data compression Codecs. Being the methods general, they can be used for specialized standard compression programs that will be developed in the future. Another main characteristic of our methods is that they require very little, or none at all, programming and knowledge of Hadoop to carry out a rather complex task. Our methods apply also to the Apache Spark framework, when used to process FASTA/Q files stored on the Hadoop File System.

We have also shown that the use of specialized FASTA/Q Hadoop Codecs, not available before this work, is advantageous in terms of space and time savings. That is, we provide effective and readily usable tools that have a non-negligible effect on saving costs in genomic data storage and processing within Big Data Technologies.

Apart from the specific recommendations provided to a potential user of the specialized compressors imported in Hadoop via our methods, this research also highlights the characteristics of specialized compressor in order to be proficuously imported in Hadoop. Namely, effective “local” compression and fast decompression time.

## Availability of data and materials

Project name: FASTdoopC

Project home page: https://github.com/fpalini/fastdoopc

Operating system(s): Platform independent

Programming language: Java

Other requirements: Java 8 or higher, Hadoop 3.1.1 or higher/Spark 2.3.3 or higher

License: Apache LicenseLicense: Apache License

Any restrictions to use by non-academics: None

A copy of the datasets analysed during the current study is available on the FASTdoopC project web site, at https://github.com/fpalini/fastdoopc.

## Supplementary information

**Additional file 1.** Supplementary Material.

## Abbreviations

CB: Compressed block
HDFS: Hadoop file system
HPC: High-performance computing
HTS: High-throughput sequencing
