# Correction to: FASTA/Q data compressors for MapReduce-Hadoop genomics: space and time savings made easy

**DOI:** 10.1186/s12859-022-04600-6

**Published:** 2022-02-15

**Authors:** Umberto Ferraro Petrillo, Francesco Palini, Giuseppe Cattaneo, Raffaele Giancarlo

**Affiliations:** 1 Dipartimento di Scienze Statistiche, Università di Roma - La Sapienza, Rome, Italy; 2 Dipartimento di Informatica, Università di Salerno, Fisciano, Italy; 3 Dipartimento di Matematica ed Informatica, Università di Palermo, Palermo, Italy

## Correction to: BMC Bioinformatics (2021) 22:144 10.1186/s12859‑021‑04063‑1

Following publication of the original article [1], the authors identified that the affiliations of Giuseppe Cattaneo and Raffaele Giancarlo were interchanged. The correct affiliations are given below.

The correct affiliation of Giuseppe Cattaneo is:

^2^Dipartimento di Informatica, Università di Salerno, Fisciano, Italy.

The correct affiliation of Raffaele Giancarlo is:

^3^Dipartimento di Matematica ed Informatica, Università di Palermo, Palermo, Italy.

The original article [1] has been corrected.
