# Supplementary material for: FASTA/Q data compressors for MapReduce-Hadoop genomics: space and time savings made easy
Source: BMC Bioinformatics. 2021 Mar 22;22:144. doi: 10.1186/s12859-021-04063-1 (PMC7986029; doi:10.1186/s12859-021-04063-1)
Supplement: Supplementary file 1 — Additional file 1. Supplementary Material. [file 12859_2021_4063_MOESM1_ESM.pdf]

FASTA/Q Data Compressors for MapReduce-Hadoop Genomics: Space and Time Savings Made Easy

Supplementary Material

Umberto Ferraro Petrillo* † Francesco Palini* Giuseppe Cattaneo‡ §
Raffaele Giancarlo¶ §

Abstract

Additional details about the Main Manuscript are provided in this document.

1 The *MapReduce* Programming Paradigm and Hadoop

1.1 The Paradigm

MapReduce [3] is a paradigm for the processing of large amounts of data on a distributed computing infrastructure. Assuming the input data is organized as a set of $<key, value>$ pairs, it is based on the definition of two functions. The *map* function processes an input $<key, value>$ pair and returns a (possibly empty) intermediate set of $<key, value>$ pairs. The *reduce* function merges all the intermediate values sharing the same key to form a (possibly smaller) set of values. These functions are run, as tasks, on the nodes of a distributed computing framework. All the activities related to the management of the lifecycle of these tasks as well as the collection of the map function results and their transmission to the reduce functions are transparently handled by the underlying framework (*implicit parallelism*), with no burden on the programmer.

1.2 Apache Hadoop

Apache Hadoop is the most popular framework supporting the *MapReduce* paradigm. It allows for the execution of distributed computations thanks to the interplay of two architectural components: YARN (*Yet Another Resource Negotiator*) [8] and HDFS (*Hadoop Distributed File System*) [7]. YARN manages the lifecycle of a distributed application by keeping track of the resources available on a computing cluster and allocating them for the execution of application tasks modeled after one of the supported computing paradigms. HDFS is a distributed and block-structured file-system designed to run on commodity hardware and able to provide fault tolerance through replication of data.

A basic Hadoop cluster is composed of a single *master node* and multiple *worker nodes*. The master node arbitrates the assignment of computational resources to applications to be run on the cluster and maintains an index of all the directories and the files stored in the HDFS distributed file system. Moreover, it tracks the worker nodes physically storing the HDFS data blocks making up these files. The worker nodes host a set of *worker*s (also called *Containers*), in charge of running the map and reduce tasks of a *MapReduce* application, as well as using the local storage to maintain a subset of the HDFS data blocks.

*Dipartimento di Scienze Statistiche, Università di Roma - La Sapienza, Rome, 00185, Italy
†To whom correspondence should be addressed.
‡Dipartimento di Informatica, Università di Salerno, Fisciano (SA), 84084, Italy
§Those two authors contributed equally to the research.
¶Dipartimento di Matematica ed Informatica, Università di Palermo, Palermo, 90133, Italy

One of the main characteristics of Hadoop is its ability to exploit *data-local* computing. By this term, we mean the possibility to move applications closer to the data (rather than the vice-versa). This allows to greatly reduce network congestion and increase the overall throughput of the system when processing large amounts of data. Moreover, in order to reliably maintain files and to properly balance the load between different nodes of a cluster, large files are automatically split into smaller HDFS data blocks, replicated and spread across different nodes.

2 Specialized Compressors Supported by means of our Splittable Compressor Meta-Codec

Among the many compression algorithms specialized for genomic data [4], DSRC is the only featuring a splittable Codec among the data compression tools achieving the best performance, based on benchmarking, when dealing with FASTA/Q files.

It represents a robust testbed for our solution because its original implementation has been developed in C++ and its integration within a Java Codec is not trivial to realize.

A DSRC standard compressed file is organized in three parts.

- **Body.** It contains a set of compressed data blocks. Each of these is compressed and can be decompressed independently from the others. The default size of each compressed data block is 10MB.
- **Header.** It reports the number of compressed data blocks existing in that file, the size of the footer and its relative position inside the file.
- **Footer.** It reports the size of each compressed data block and the flags used for its compression.

2.1 Implementation details

The special-purpose Codec supporting DSRC, HS_DSRC, has been obtained following our **Splittable Compressor Meta-Codec**, as described in Section 2.3 of the Main Manuscript. It required the development of two Java classes: `DSRCInputFormat` and `DSRCCodec`. In particular, `DSRCCodec` uses the JNI framework[5] to load in memory and instantiate the dynamic library containing the DSRC native implementation. Then, it uses the `DSRCInputFormat` class to extract the information regarding the DSRC parameters and the list of compressed data blocks, according to the DSRC format. In addition, this class initializes the `CodecInputStream` object, pointing to the file to be decompressed during the execution of a job. Finally, it runs the `NativeCodecDecompressor decompress` method on each compressed data block to obtain its decompressed version.

3 Specialized Compressors Supported by means of our Universal Compressor Meta-Codec

In this Section we provide details about the work done for incorporating in Hadoop the specialized compressors reported in Section 2.5.1 of the Main Manuscript, using our **Universal Compressor Meta-Codec**.

For each compressor, the only step required to support it is the definition of a set of properties stating the supported input file types and the command-line required for compressing and decompressing a generic input file. Let X be the unique name denoting the compressor to be supported and F the file being processed, the following command line properties are available for its integration:

- `uc.X.compress.cmd`: the command line to be used for compressing F using X.
- `uc.X.decompress.cmd`: the command line to be used for decompressing F using X.
- `uc.X.io.input.flag`: the command line flag used to specify the input filename.

Properties	Compressors				
	SPRING (for FASTQ)	SPRING (for FASTA)	DSRC	FqzComp	MFCompress
uc.X.compress.cmd	spring -c	spring -c –fasta-input	dsrc c -t8	fqz_comp	MFCompressC -t 8 -p 8
uc.X.decompress.cmd	spring -d	spring -d	dsrc d -t8	fqz_comp -d	MFCompressD -t 8
uc.X.io.input.flag	-i	-i			
uc.X.io.output.flag	-o	-o			-o
uc.X.compress.ext	.spring	.spring	.dsrc	.fqz	.mfc
uc.X.decompress.ext		.fasta			.fasta
uc.X.io.reverse					true

Table 1: Command line properties required for supporting several specialized compressors using our **Universal Compressor Meta-Codec**

- `uc.X.io.output.flag`: the command line flag used to specify the output filename.
- `uc.X.compress.ext`: the extension used by X for saving a compressed copy of F.
- `uc.X.decompress.ext`: the extension used by X for saving a decompressed copy of X ("fastq" by default).
- `uc.X.io.reverse`: if X requires the output file name to be specified before the input file name, it is set to *true*. false, otherwise.

In Table 1, the command lines used for integrating the target specialized compressors using our **Universal Compressor Meta-Codec** are reported.

4 Datasets

For our experiments we considered two different datasets.

The first type of dataset, referred to as **type 1 datasets**, is a collection of FASTQ and FASTA files of different sizes. The FASTA files of these datasets contain a set of reads extracted uniformly at random from a collection of genomic sequences coming from the Human genome [1]. The FASTQ files of these datasets contain a set of reads extracted uniformly at random from a collection of genomic sequences coming from the Pinus Taeda genome [9]. Details about these datasets are reported in Table 2 and Table 3.

Name	# of reads	Avg. read length
16GB	96,407,378	100
32GB	192,653,438	100
64GB	385,306,876	100
96GB	577,960,314	100

Table 2: **Type 1 datasets:** details about FASTA files included in these datasets.

Name	# of reads	Avg. read length
16GB	44,681,859	151
32GB	89,363,718	151
64GB	178,727,437	151
96GB	268,091,154	151

Table 3: **Type 1 datasets:** details about FASTQ files included in these datasets.

The second type of dataset, referred to as **type 2 datasets**, is a collection of FASTQ files corresponding to different coverages of the *H.sapiens* genome. It has been assembled using the same methodology and the

same input FASTQ files considered in [2] for their experiments: that is, more input FASTQ files with a known coverage are concatenated to get an higher coverage.

Namely, the hsapiens1 dataset (coverage 1.6x) has been obtained by concatenating the SRR062634_1.fastq and SRR062634_2.fastq files. The hsapiens2 dataset (coverage 14.4x) has been obtained by concatenating the ERP174324_1.fastq and ERP174324_2.fastq files. The hsapiens3 dataset (coverage 26.6x) has been obtained by concatenating the NA12878-Rep-1_S1_L001_R1_001.fastq and NA12878-Rep-1_S1_L001_R1_002.fastq files. The only difference with respect to the methodology used in [2] is that we had not to first trim input sequences because our HS and HU Codecs support variable length reads.

Details about these datasets are reported in Table 4.

Name	# of reads (M)	Avg. read length	Coverage	Originating Files
hsapiens1	48.9	100	1.6x	SRR062634_1.fastq, SRR062634_2.fastq
hsapiens2	447.1	101	14.4x	ERP174324_1.fastq, ERP174324_2.fastq
hsapiens3	560.4	149 (var. length)	26.6x	NA12878-Rep-1_S1_L001_R1_001.fastq, NA12878-Rep-1_S1_L001_R2_001.fastq

Table 4: **Type 2 datasets:** details about FASTQ files included in these datasets.

5 Assessing the invariance of the compression properties of codecs executed via our HU and HS Codecs

In order to prove that our HU and HS Codecs do not change in any way the compression properties of the compressors we import, we perform the following experiment. We create two $128MB$ input files (equivalent to one single HDFS block in our Hadoop installation) by extracting the corresponding number of bytes from the initial part of the 16GB FASTA file and of the 16GB FASTQ file of our **type 1 datasets**. Then, we compress the outcoming files, using each of the considered FASTA/Q specialized compression codecs, in their original form, as well as the same codecs, as imported in our HU Codec and in our HS Codec (when available). At this point, we check if the two files asre identical. A simple but effective way to perform this check is by compare the MD5 hashes of the two files. MD5 is an hash function used in cryptography to produce a 128-bit message digest so that the probability for two different files to generate the same hashes is extremely low (see [6]). So, we assessed that the compressed file returned by each codec was identical to the one returned by the same codec encapsulated in our HU Codec and our HS Codec by comparing the corresponding MD5 hashes, as reported in Tables 5-6.

Codec	Stand-alone	Executed via HS	Executed via HU
BZIP2	12e952e997173ca2cfe8b27522c72ac9	12e952e997173ca2cfe8b27522c72ac9	-
LZ4	7cce35d0c71a96f1b65d371a26a6497d	7cce35d0c71a96f1b65d371a26a6497d	-
ZSTD	846daee9b75914ce017e39e3f1a48502	846daee9b75914ce017e39e3f1a48502	-
MFCompress	b36ef77d35a52617e5c776b0b33bde95	-	b36ef77d35a52617e5c776b0b33bde95
SPRING	375d61ccbd0e7826805e7f95004241ba	-	375d61ccbd0e7826805e7f95004241ba

Table 5: MD5 hashes of the compressed files obtained by executing compression codecs either as stand-alone methods or as an encapsulation of our HS and HU Codecs using, as input, the first 128MB of the 16GB FASTA file of our **type 1 datasets**.

Codec	Stand-alone	Executed via HS	Executed via HU
BZIP2	4e7ce591e442165ba6498462141c597b	4e7ce591e442165ba6498462141c597b	-
LZ4	407158640e657d993235443ce8b792dc	407158640e657d993235443ce8b792dc	-
ZSTD	0931574e2b438471908c1edef76fac31	0931574e2b438471908c1edef76fac31	-
DSRC	aac9334e75df250647a197ac0a0d68bc	aac9334e75df250647a197ac0a0d68bc	aac9334e75df250647a197ac0a0d68bc
Fqzcomp	68aed4051105fbdedadb23935da05245	-	68aed4051105fbdedadb23935da05245
SPRING	6ad1e04dd15e2002f52b69c68ee477c2	-	6ad1e04dd15e2002f52b69c68ee477c2

Table 6: MD5 hashes of the compressed files obtained by executing compression codecs either as stand-alone methods or as an encapsulation of our HU and HS codecs using, as input, the first 128MB of the 16GB FASTQ file of our **type 1 datasets**.

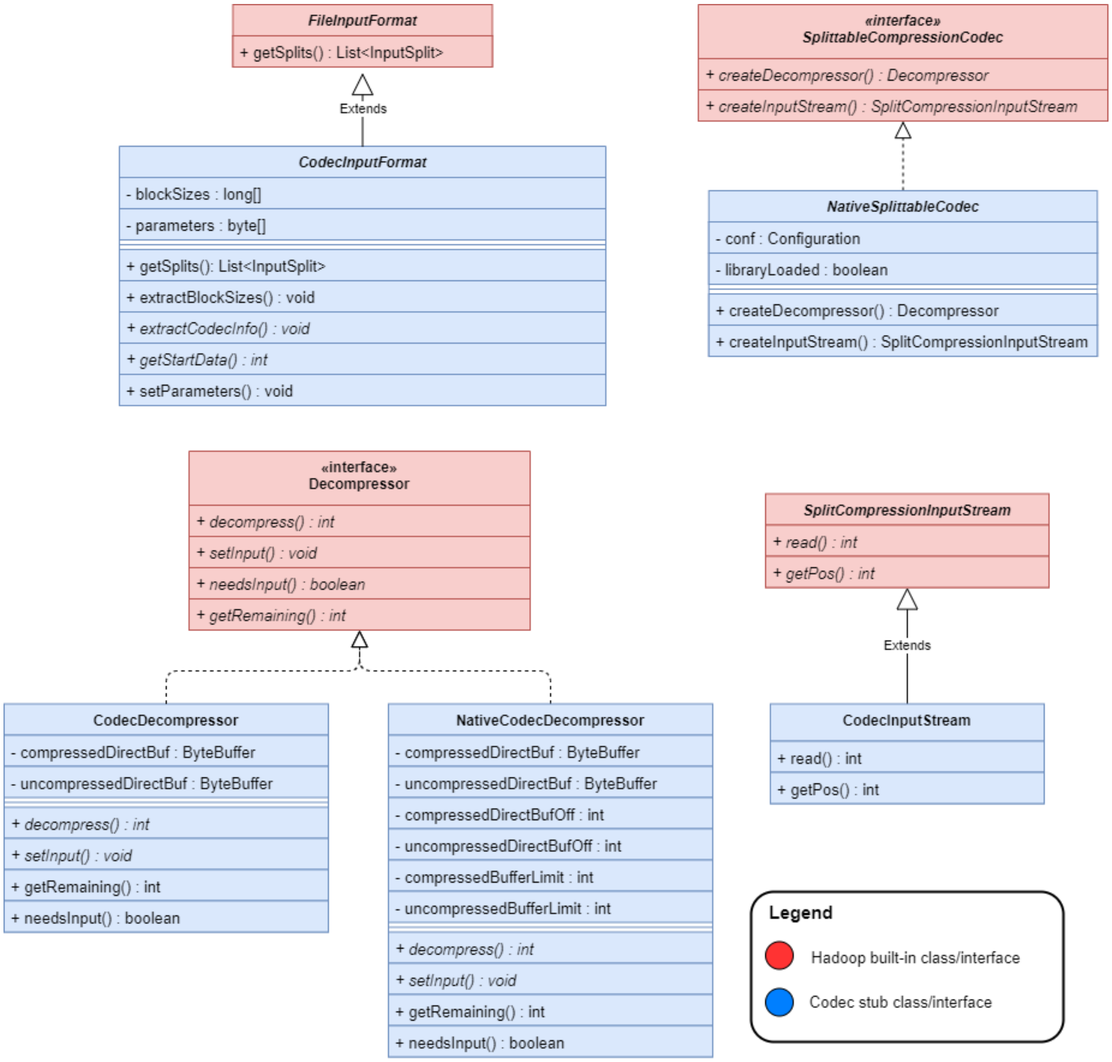

Figure 1: UML class diagram of our **Splittable Compressor Meta-Codec**

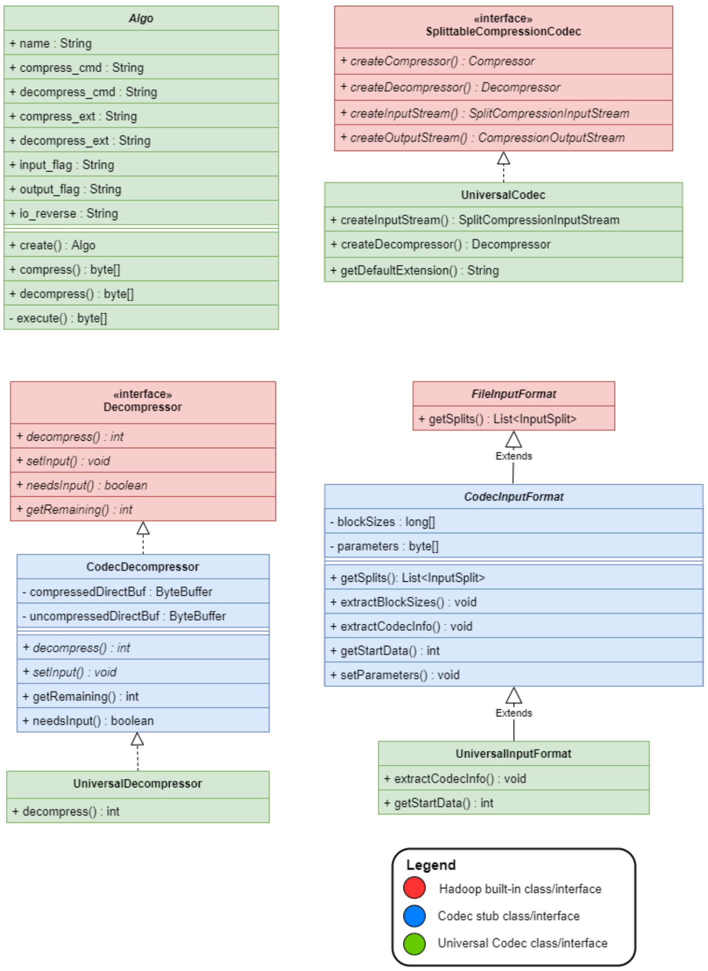

Figure 2: UML class diagram of our **Universal Compressor Meta-Codec**

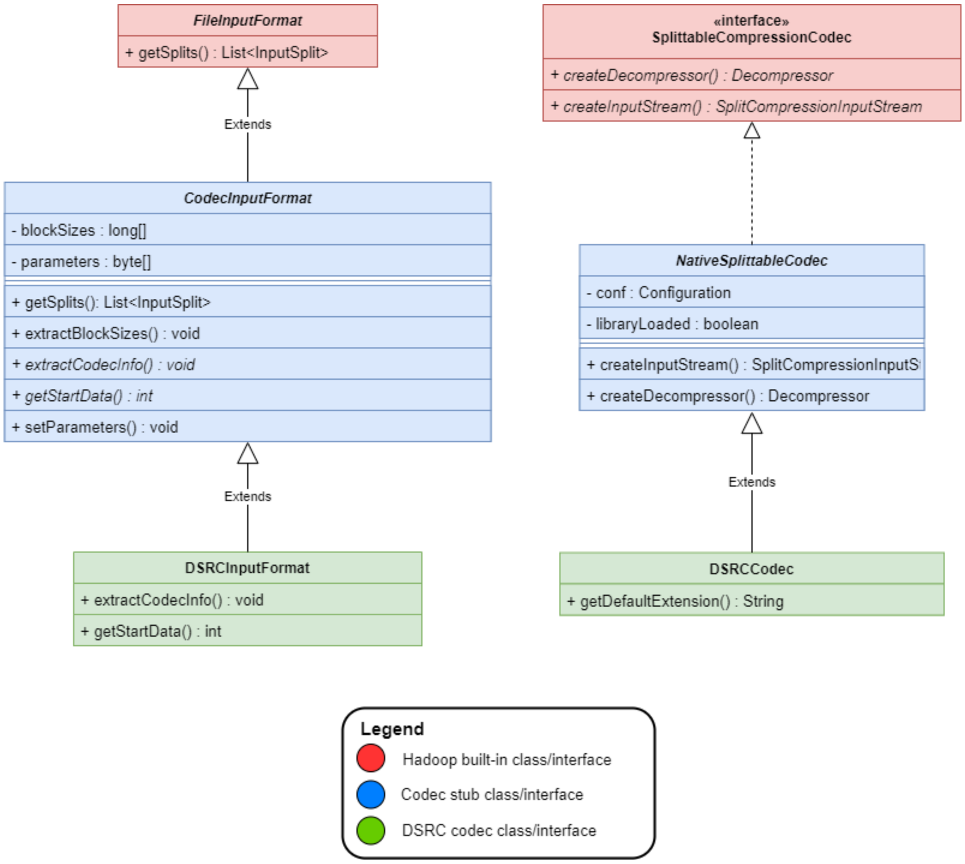

Figure 3: UML class diagram of HS_DSRC

References

[1] D. R. Bentley, S. Balasubramanian, H. P. Swerdlow, G. P. Smith, J. Milton, C. G. Brown, K. P. Hall, D. J. Evers, C. L. Barnes, H. R. Bignell, et al. Accurate whole human genome sequencing using reversible terminator chemistry. *Nature*, 456(7218):53–59, 2008.

[2] S. Chandak, K. Tatwawadi, I. Ochoa, M. Hernaez, and T. Weissman. SPRING: a next-generation compressor for FASTQ data. *Bioinformatics*, 35(15):2674–2676, 2019.

[3] J. Dean and S. Ghemawat. MapReduce: simplified data processing on large clusters. *Communications of the ACM*, 51:107–113, 2008.

[4] I. Numanagi˜c, J. K. Bonfield, F. Hach, J. Voges, J. Ostermann, C. Alberti, M. Mattavelli, and S. C. Sahinalp. Comparison of high-throughput sequencing data compression tools. *Nature Methods*, 13:1005–1009, 2016.

[5] Oracle. Java Native Interface Specification. (Available from: `https://docs.oracle.com/javase/7/docs/technotes/guides/jni/spec/jniTOC.html`), 1998.

[6] R. Rivest. Rfc1321: The md5 message-digest algorithm, 1992.

[7] K. Shvachko, H. Kuang, S. Radia, and R. Chansler. The Hadoop distributed file system. In *Proceedings of the 2010 IEEE 26th Symposium on Mass Storage Systems and Technologies (MSST)*, MSST '10, pages 1–10, Washington, DC, USA, 2010. IEEE Computer Society.

[8] V. K. Vavilapalli, A. C. Murthy, C. Douglas, S. Agarwal, M. Konar, R. Evans, T. Graves, J. Lowe, H. Shah, S. Seth, et al. Apache Hadoop YARN: Yet another resource negotiator. In *Proceedings of the 4th annual Symposium on Cloud Computing*, page 5. ACM, 2013.

[9] J. L. Wegrzyn, B. Y. Lin, J. J. Zieve, W. M. Dougherty, P. J. Martinez-Garcia, M. Koriabine, A. Holtz-Morris, P. deJong, M. Crepeau, C. H. Langley, D. Puiu, S. L. Salzberg, D. B. Neale, and K. A. Stevens. Insights into the loblolly pine genome: characterization of BAC and fosmid sequences. *PLoS One*, 8(9):e72439, 2013.
